# Impacts of Anthropogenic Changes on the Mun River Water: Insight from Spatio-Distributions and Relationship of C and N Species in Northeast Thailand

**DOI:** 10.3390/ijerph16040659

**Published:** 2019-02-23

**Authors:** Jinke Liu, Guilin Han, Xiaolong Liu, Man Liu, Chao Song, Qian Zhang, Kunhua Yang, Xiaoqiang Li

**Affiliations:** 1 School of Scientific Research, China University of Geosciences (Beijing), Beijing 100083, China; Liujinke@cugb.edu.cn (J.L.); lman@cugb.edu.cn (M.L.); zhangqian9@cugb.edu.cn (Q.Z.); Kunhuayang@cugb.edu.cn (K.Y.); lxq166@cugb.edu.cn (X.L.); 2 Tianjin Key Laboratory of Water Resources and Environment, Tianjin Normal University, Tianjin 300387, China; xiaolong.liu@tjnu.edu.cn; 3 The Institute of Hydrogeology and Environmental Geology, Chinese Academy of Geological Sciences, Shijiazhuang 050061, China; songchao@mail.cgs.gov.cn; 4 Institute of Geographic Sciences and Natural Resources Research, Chinese Academy of Sciences, Beijing 100101, China

**Keywords:** dissolved organic carbon, total dissolved nitrogen, agricultural land-use, water quality, Mun River, northeast Thailand

## Abstract

C and N species, including dissolved organic carbon (DOC), dissolved inorganic carbon (DIC), dissolved organic nitrogen (DON), NO_3_^−^ and NH_4_^+^ contents in 57 river water samples collected from the Mun River of Thailand were measured to determine the relationships between these dissolved load species and their impacts on the environment. DOC values varied between 1.71 and 40.08 mg/L, averaging 11.14 mg/L; DON values ranged from 0.20 to 1.37 mg/L, with an average value of 0.48 mg/L; NO_3_^−^-N values averaged 0.18 mg/L; and NH_4_^+^-N values averaged 0.15 mg/L. DOC contents increased while DON and NO_3_^−^ values decreased along the flow direction. The concentrations of NH_4_^+^ maintained the same level in the whole watershed. DOC and DON values exhibited clearly higher concentrations in comparison with other rivers worldwide and were inextricably linked with anthropogenic inputs. The relationships of DOC, DON, and anthropogenic ions imply that there are two different anthropogenic sources (industrial activities and agricultural activities) of the dissolved load in the Mun River watershed. The limited correlations between the DON, NO_3_^−^, and NH_4_^+^ indicate that the N species are not dominated by a single factor, and reciprocal transformations of riverine N pool are complex. Based on the environmental water quality standard reported by the EC (European Communities) and the World Health Organization, assessments of the water quality using the parameters of pH, dissolved oxygen (DO), NO_3_^−^, NH_4_^+^, and TN (total nitrogen) in the Mun River were conducted. The results demonstrate that the river water faces potential environmental pollution, and anthropogenic inputs endanger local water quality and the aquatic community. Therefore, the local government should restrict and reduce the anthropogenic inputs discharged in to rivers, and launch long-term monitoring of water quality.

## 1. Introduction

Riverine total dissolved carbon (TDC, including dissolved inorganic carbon and dissolved organic carbon) and total dissolved nitrogen (TDN, including dissolved inorganic nitrogen and dissolved organic nitrogen), as omnipresent materials in the hydrosphere, have inextricable associations with water quality, and can reflect the degree of contamination of river waters [1,2,3]. Dissolved organic carbon can influence the acidity of aquatic ecosystems [4], and increase the capacity of metal transport with the formation of organic complexes [5,6], which may result in the enrichment of toxic metals in aquatic biotas [7]. Moreover, dissolved organic carbon (DOC) makes up nearly 50% of the carbon flux exported to the sea [8] and is an important component of the global carbon cycle. Thus, DOC is closely linked to climate and environmental changes [9,10,11]. TDN is an important index to assess water quality. Excessive nitrogen inputs can disturb the functioning of the energy and material exchanges in the riverine community [12,13], and finally lead to eutrophication [14]. In addition, TDN can interfere with drinking water treatment processes by reducing chlorine levels and producing carcinogenic organo-chlorine compounds which can cause damage to humans [15,16]. Therefore, studies of TDC and TDN contents in the river waters at the watershed scale have significant environmental significance in evaluating the turnover capacity of C, N species and the health of rivers.

Riverine DOC and TDN mainly originate from soil leaching [17,18], complicated biological processes (e.g., the production and release of bacterial and phytoplankton, zooplankton feeding [19,20,21]), and anthropogenic inputs [22]. Previous studies [23,24,25] highlighted that both carbon and nitrogen fluxes have been substantially increasing over past decades as a response to the environment and climate change. Human activity plays an important role in this process [26,27]. On the one hand, anthropogenic inputs such as domestic sewage, industrial effluents, fertilizers, and irrigation can directly increase the concentrations of riverine carbon and nitrogen [28,29]. On the other hand, human activities have ecological impacts [1]. At the watershed scale, land-use conversion may alter physicochemical parameters, landscapes, and hydrological cycles [30,31,32], which are understood to be important factors relating to TDC and TDN [33,34]. However, as a result of human activities on the global scale, the abnormal changes of climate and temperature also have an impact on the carbon and nitrogen species [35,36]. Thus, full assessments of TDC and TDN distributions and relationships in watersheds, particularly with respect to influences by human activity, are urgently required.

The Mun River Basin is the largest river basin in Thailand. Industrial development and long-term agricultural activities such as the fertilizer application and irrigation may bring about various forms of environmental impact and pollution [37,38]. However, few studies have documented water quality and pollution in Mun River water. The aims of this study are: (1) to evaluate the variations of C and N species concentrations at the watershed scale; (2) to investigate the relationship between the C and N species and identify the possible geochemical processes; (3) to analyze the impacts of anthropogenic inputs on the dissolved load species of river water; and (4) to assess the water quality of river water.

## 2. Study Area and Method

### 2.1. Study Area Description and Sampling Procedure

**Figure 1:**
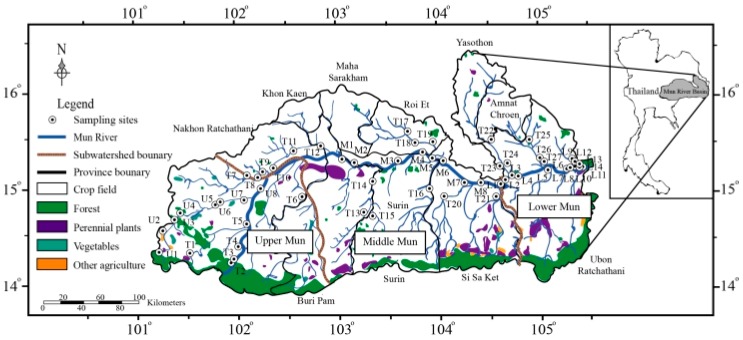
Location, land-use type and sampling sites in the Mun River Basin.

The Mun River, as the tributary of Mekong River, originates from Nakhon Ratchasima province, flows through 10 provinces, and joins the Mekong River in the Ubon Ratchathani province (Figure 1). It has a drainage area of 71,060 km^2^ (14° N–16° N and 101°30′ E–105°30′ E), and a total length of 673 km. The annual water discharge is about 2.5 × 10^9^ m^3^ [37]. The Mun River Basin has a tropical savannah climate [39], leading to high rainfall from May to October with an annual precipitation between 800 and 1800 mm. The mean air temperature is from 25 to 30 °C. According to previous studies [37], the whole watershed is often divided into three sub-watersheds: the Upper Mun (101°30′ E–102°30′ E), the Middle Mun (102°30′ E–104°30′ E), and the Lower Mun (104°30′ E–105°30′ E).

A total of 57 water samples were collected in Mun river in July 2017. The locations of sampling sites are illustrated in Figure 1. All the water samples were collected at a depth about 10 cm, and each container was rinsed three times with the corresponding sample. The samples for DOC and DON analysis were immediately filtered through a 0.22-μm Millipore filter (Whatman GF/F, pre-cleaned, General Electric Company (GE), Boston, MA, USA) and stored in the high-density polyethylene (HDPE) bottles that had been pre-cleaned. The samples were then acidified with ultra-purified HCl to keep pH < 2. The samples for ions analysis were directly stored in the HDPE bottles. All collected samples were and kept refrigerated at approximately 4 °C prior to analysis.

### 2.2. Study Area Description and Sampling Procedure

The physicochemical parameters of temperature, pH, Total dissolved solids (TDS), and dissolved oxygen (DO) were measured in situ using the water quality analyzer YSI-6920 (Xylem Inc., Yellow Springs, OH, USA). HCO_3_^−^ was titrated by HCl (0.03 M) in the field, ions (Cl^−^) were detected by the ion chromatograph ICS-900 (DIONEX, Sunnyvale, CA, USA), and N species (NO_3_^−^, NH_4_^+^) were determined by using a Continuous Flow Analyzer (AA3, SEAL Analytical GmbH, Norderstedt, Germany) at Tianjin Normal University. The DOC and DON values were determined by Elementar Vario TOC (Elementar, Hanau, Germany) at the Laboratory of Surficial Environmental Geochemistry, China University of Geosciences (Beijing). Replicate samples were employed to achieve the accuracy of the analysis. The precisions of all the analyses were better than ±5%.

All the data were processed with ArcView GIS software (ESRI., Redlands, CA, USA), SPSS 19.0 software (SPSS Inc., Chicago, IL, USA) and the figures were completed with Origin 8.0 (Origin Lab., Hampton, MA, USA) and Adobe Illustrator (Adobe Inc., San Jose, CA, USA).

### 2.3. Land-Use Type

**Figure 2:**
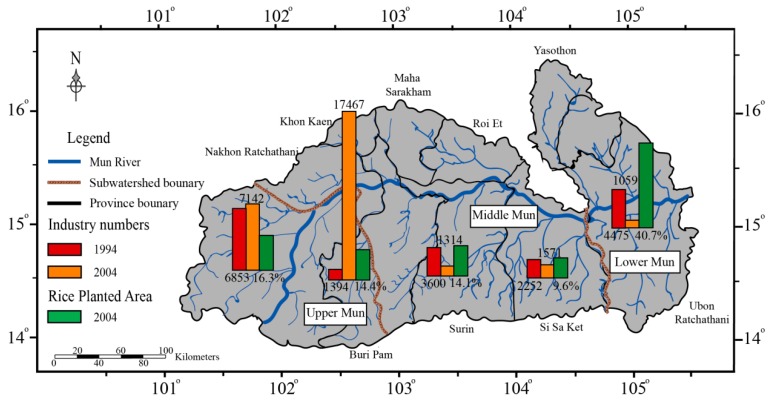
Percentage of industries and rice field in the Mun River Basin.

Thailand is one of the largest crop-producing countries [40]. In the Mun River Basin, the proportion of planted area has risen rapidly during the recent decades, specifically, the agricultural land took up 38% of the total area in 1994, 45% in 2004, and increased to nearly 80% in 2018 [37,40,41]. In addition, agricultural land can be divided into crop fields, vegetable fields, and other agricultural land, of which rice fields dominate (Figure 1). The rice fields in the Lower Mun account for a larger proportion than the Upper Mun (Figure 2). July is the vegetative stage of the crops in the Mun River Basin, which means more chemical fertilizers are applied [40]. A previous study [38] showed that agricultural activities resulted in overloading of soil nutrients, afterwards influencing the water quality in the Lower Mun. Moreover, with the development of industry over decades, factories are grouped in the Upper Mun (Figure 2), representing potential pollutant sources. Thus, it is crucial to investigate the water quality in the Mun River

## 3. Results and Discussion

### 3.1. Physicochemical Parameters

The physicochemical parameters and concentrations of C, N species are shown in Table S1. The water temperature of the Mun River varied from 20.3 to 31.1 °C, with an average of 28.7 °C. The pH value ranged from 6.4 to 8.4, with an average of 7.1, which means approximately 90% of the inorganic carbon is present as bicarbonate. TDS values ranged from 9 to 998 mg/L (with an average of 99 mg/L). Dissolved oxygen (DO) values varied from 2.9 to 7.9 mg/L (with an average of 4.9 mg/L).

DO distribution can reflect the local organic pollution processes, which have a marked impact on aquatic biota [42]. In contrast to a previous study [43], DO did not exhibit a significant correlation with the other parameters, DOC and DON, which may indicate that the hydrochemical characteristics are complex. Previous studies observed a positive relationship between DOC contents and increasing pH in the unpolluted regions which dominated by the biological effect such as photosynthesis and respiration [21,44], whereas, our field data present contradicting results. pH showed a weak but significant negative correlation with DOC (*r* = −0.43, *p* < 0.05; Table 1), which may indicate that the regional biological processes were disturbed by the anthropogenic inputs.

### 3.2. C and N Species Distributions and Relationships

**Figure 3:**
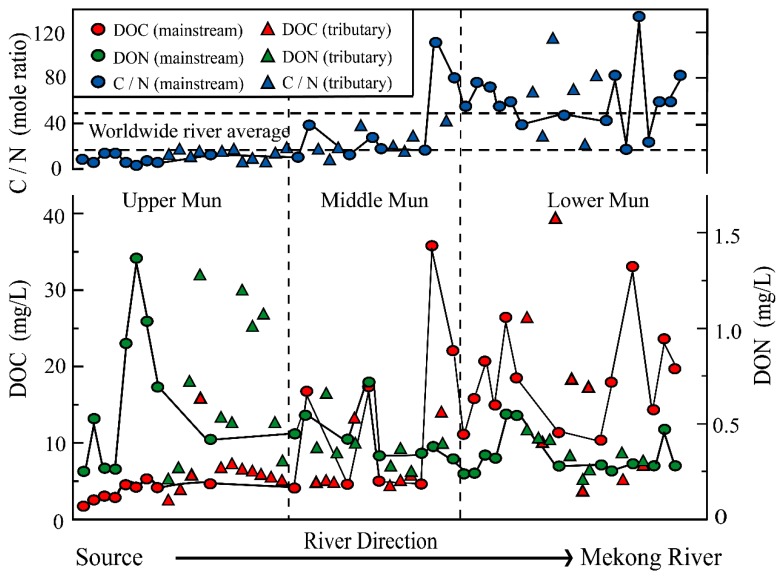
Spatial distributions of DOC, DON, and the DOC/DON mole ratio in the Mun river.

The C and N species distributions are illustrated in Table 2. Generally, the concentrations of DOC increased while DON decreased along the flow direction (Figure 3). It is noteworthy that DOC contents had large differences (from 1.71 to 40.08 mg/L) in different sampling sites.

Though biological mechanisms such as phytoplankton exudation and bacteria production are considered to be important sources of riverine DOC [45], based on regional analyses [1,20,46] it is hard to attribute such high concentrations to the biological effects, because apart from the soil-leaching processes from wetlands (peatlands) and anthropogenic inputs, riverine DOC concentrations seldom exceed 15 mg/L [26,47,48]. Since no other natural DOC sources could be addressed, the extremely high contents of DOC in the Mun River should be closely associated with anthropogenic inputs. Additionally, since the relatively large proportion of agricultural terrain and few industrial existing in the Lower Mun, the agricultural activities were more likely the reason of DOC overloading. Actually, this result is consistent with the previous studies which found that farm works are a direct source of water pollution in the Lower Mun [37,38]. NO_3_^−^ and NH_4_^+^, as common inorganic pollutants, are subject to legislative requirements and limits on concentrations. In the Mun River watershed, NO_3_^−^ values varied from 0.01 to 1.06 mg/L, with an average of 0.18 mg/L, while NH_4_^+^ values averaged 0.15 mg/L, ranging between 0.10 and 0.29 mg/L, respectively. Concentrations of the inorganic nitrogen were relatively lower in comparison to DON, whereas NO_3_^−^ contents were obviously higher in the Upper Mun, which may imply the industrial inputs [49].

**Figure 4:**
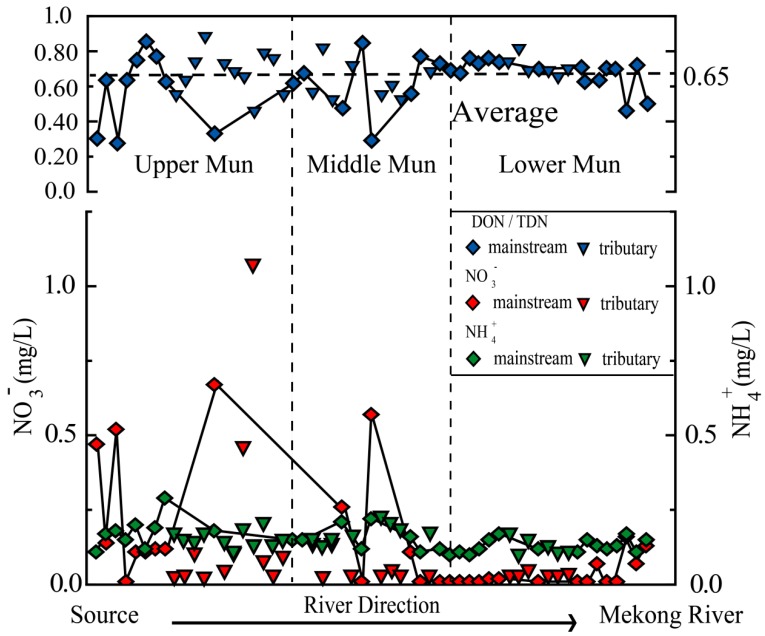
Spatial distributions of NO_3_^−^, NH_4_^+^, and the DON/TDN mole ratio in the Mun river.

**Figure 5:**
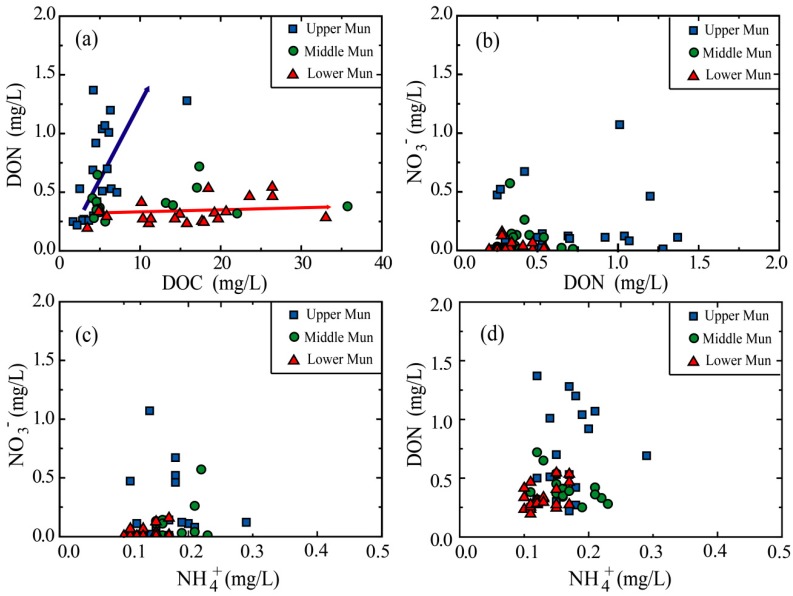
Correlations between the DOC and species: (**a**) DOC vs. DON; (**b**) DON vs. NO_3_^−^; (**c**) NH_4_^+^ vs. NO_3_^−^; (**d**) NH_4_^+^ vs. DON.

DON comprised the majority of TDN in Mun River, accounting for 27.5–88.8% (average 64.8%) of the TDN, (Figure 4), consistent with dominance of DON in the riverine N pool [50,51]. Some studies maintained that the observed high DON/TDN percentage may result from the biological effect: phytoplankton and microorganisms uptake the dissolved NO_3_^−^ and NH_4_^+^, undergo series of complex biological processes, and then release DON through cell disruption and passive exudation [20,52]. In fact, the bacterial ammonification and nitrification can convert DON to NO_3_^−^ and NH_4_^+^ conversely [53], and thus it is quite hard to discern which biological process dominates the reciprocal transformation of the N species. In contrast to some regional analyses which attribute turnover of nitrogen biogeochemical cycle to the biological effect and have significant linear correlation between the DON, NO_3_^−^, and NH_4_^+^ [24,54,55], there are no clear linear correlations between the N species in the Mun River (Figure 5, Table 2), indicating that both the local biogeochemical processes and the anthropogenic activities inputs play important roles in the Mun River.

DOC showed strong correlations with DON in the Upper Mun and Lower Mun, respectively (Figure 5), which may imply DOC and DON have same sources at each sub-watershed. Previous study [56] reported an average ratio of DOC/DON of about 32.5 ± 16.3 for rivers worldwide. As shown in Figure 3, DOC/DON ratios in the Upper Mun are lower than the average value in rivers worldwide, while in the Lower Mun, DOC/DON ratios are clearly higher than the average values worldwide (Figure 3). Since the relative variation degrees of DOC were much larger than for DON, the ratios of DOC/DON observed in this study mainly depend on the DOC concentrations and result from anthropogenic inputs. In fact, the similar abnormal ratios have also been observed where the anthropogenic contaminations are serious [14,21,24]. Moreover, based on the industrial and agricultural terrain distribution in the Mun River Basin (Figure 2); more specifically, industrial terrain is concentrated in the Upper Mun, while the agricultural area is concentrated in the Lower Mun. The distinct DOC/DON ratios distributed in the upstream and downstream may result from different anthropogenic sources.

### 3.3. Atmospheric Inputs

**Figure 6:**
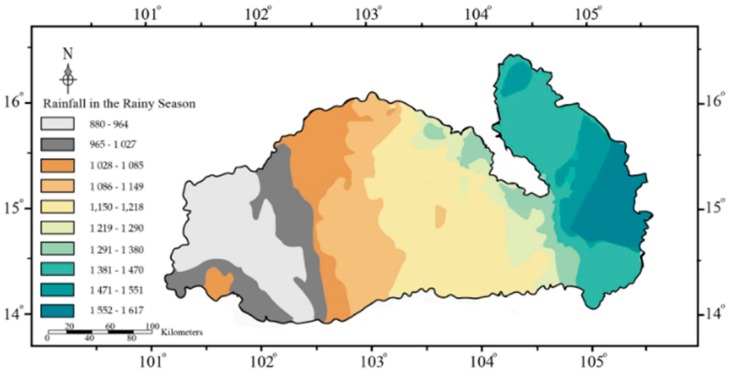
Spatial patterns of annual precipitation in the wet season in the Mun River Basin.

The Mun River Basin has a wet season (May–October) and a dry season (November–April). A previous study [38] collected long-term precipitation observational data from 1960 to 2015. The annual average rainfall in the wet season is 10 times higher than that in dry season, and river flow is mainly from runoff of watershed in the rainfall period [37]. Spatial distribution of precipitation in rainfall period is shown in Figure 6; it is clear that the precipitation increases along the flow direction. Since the DOC and DON contents in the rainfall are negligible [12,16], the high rainfall will dilute the concentrations of DOC and DON. However, the Lower Mun, which has higher rainfall, shows higher DOC contents (Table 2), demonstrating that the variations of DOC cannot be attributed to the rainfall; there are other sources of DOC in the Lower Mun (discussed in the next section). The variations of DON and NO_3_^−^ seem to be in accordance with the dilution effect (high concentrations in low rainfall areas); however, the high contents are concentrated in several sampling sites in the Upper Mun, which faces industrial pollution, and the concentrations in remaining sites keep the same level. This does not exclude the occurrence of dilution effect, but the distributions of the DON and NO_3_^−^ are more likely be related to the anthropogenic inputs (discussed in the next section).

### 3.4. Anthropogenic Inputs

To investigate the influence of anthropogenic input to dissolved solutes in river waters, anthropogenic ions must also be considered. The common perception is that riverine NO_3_^−^ and Cl^−^ are derived mainly from human activities [57,58]:

[Cl^−^]_riv_ = [Cl^−^]_atm_ + [Cl^−^]_anthro_ + [Cl^−^]_evap_ (1)

[NO_3_^−^]_riv_ = [NO_3_^−^]_atm_ + [NO_3_^−^]_anthro_ (2)

where riv = river; atm = atmosphere; anthro = anthropogenic inputs; and evap = evaporite weathering input.

The total amount of evaporites in the Mun River Basin is negligible, so the contribution of evaporites was ignored in this study. Samples with the lowest Cl^−^ and NO_3_^−^ contents were assumed to represent the atmospheric inputs, and the remaining Cl^−^ and NO_3_^−^ were derived from anthropogenic inputs [59]. Cl^−^ values varied from 1.6 to 603.8 mg/L, with an average of 34.6 mg/L, Given that the assumption is correct, most of the dissolved Cl^−^ may be closely related to anthropogenic inputs. Similar to NO_3_^−^, Cl^−^ exhibited clearly higher concentrations in the Upper Mun (ranging from 2.2 to 603.8 mg/L, average 68.9 mg/L) than the Lower Mun (varied between 1.6 and 31.3 mg/L, averaged 12.5 mg/L). The Cl^−^ concentrations of sampling sites T7–T11 located in the Upper Mun varied from 109.9 mg/L to 603.8 mg/L, with Cl^−^/HCO_3_^−^ values greater than 1, showing a clear impact of anthropogenic inputs. Based on the previous studies [57,58,59] and the land-use type in Mun River Basin, the high Cl^−^ and NO_3_^−^ contents in the Upper Mun should come from industrial inputs.

**Figure 7:**
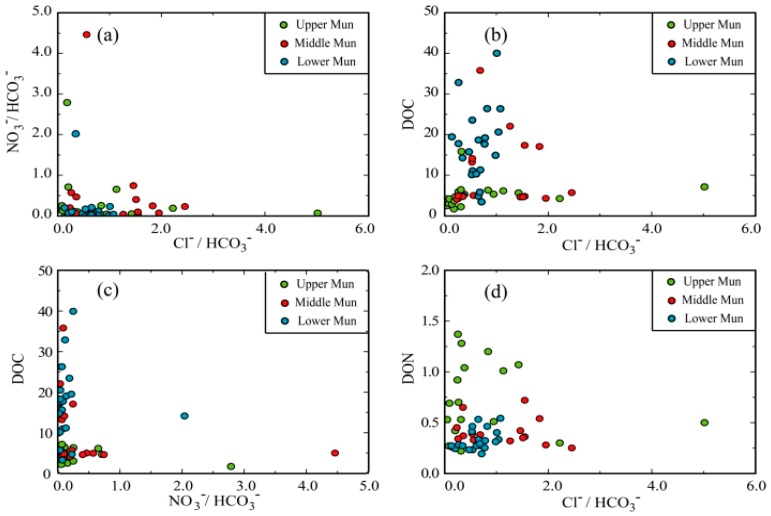
Correlations between the ions and DOC, DON: (**a**) Cl^−^/HCO_3_^−^ vs. NO_3_^−^/HCO_3_^−^; (**b**) Cl^−^/HCO_3_^−^ vs. DOC; (**c**) NO_3_^−^/HCO_3_^−^ vs. DOC; (**d**) Cl^−^/HCO_3_^−^ vs. DON.

As showed in Figure 7, the ratio of NO_3_^−^/HCO_3_^−^ and Cl^−^/HCO_3_^−^ exhibited no clear correlations in the Mun River Basin. However, it is unreasonable to deny the relationships between them, because they commonly follow same trend due to pollution [58]. The uncoupling between NO_3_^−^ and Cl^−^ in the Mun River Basin may due to two reasons: the relatively low concentrations of NO_3_^−^ in the Lower Mun and riverine nitrogen biogeochemical processes. The biological effect may generate relatively large variations of NO_3_^−^ and disturb the original ratio between NO_3_^−^ and Cl^−^. There were no correlation between DOC vs. Cl^−^/HCO_3_^−^. As mentioned before, Cl^−^ is mainly derived from industrial inputs while DOC comes from the agricultural input; the uncoupling of DOC and Cl^−^ results from the different source. DOC vs. NO_3_^−^/HCO_3_^−^ and DON vs. Cl^−^/HCO_3_^−^ also showed no correlations. As previously mentioned, the complex local biogeochemical processes may disturb the original ratios of different components in exogenous inputs, thus it is not easy to find out the definite relationship between DOC, TDN and anthropogenic inputs. Nevertheless, the connections among these dissolved loads are objective facts [25,60]; the high concentrations of these forms of organic matter and the abnormal C/N ratios are correlated with human activity. Assessments of DOC and DTN concentrations can be conducted as per legislative requirements even if the sources are still unclear.

### 3.5. Water Quality Assessments

**Figure 8:**
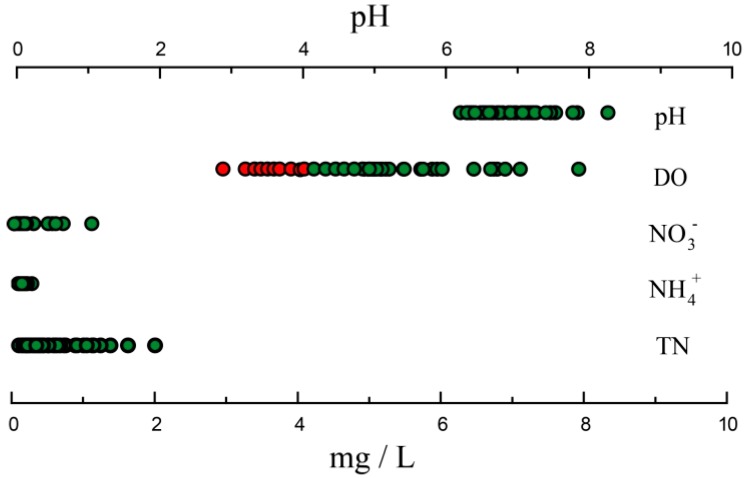
Water quality assessment of pH, DO, NO_3_^−^, NH_4_^+^, and TN in Mun River water. Based on the WHO and EC standard (pH, NO_3_^−^ for the WHO standard and DO, NH_4_^+^, TN for the European Communities (EC) standard), the green point refers to water with good quality, the red point represents polluted water.

Based on the Guidelines for Drinking Water Quality, WHO (2011, fourth edition) and EC drinking water standards (Directive 80/778/EEC) [61,62], some of water quality parameters (pH, DO, NO_3_^−^, NH_4_^+^, and TN) are shown in Figure 8. The green point indicates water with good quality that can be used for domestic and recreational purposes after suitable treatment, while the red point represents polluted water which can only be used as industrial water after treatment. A large percentage of the river water DO values is unqualified, which can influence function of aquatic biota. However, the DO decline may result from the excessive organic inputs [42], demonstrating that organic pollution in Mun river occurred, which may lead to methemoglobinemia and stomach cancer in humans via food chain and eutrophication in aquatic organisms [14,63]. N species in river waters are within standards. DOC consist of diverse organic materials such as methane, formaldehyde, benzene and so on, each pollutant have its own standard, whereas there is not an accurate standard for DOC. Thus, the worldwide river DOC and DON data were collected in this paper for comparison.

As shown in Table S2, while DON levels in rivers worldwide are variable, there are always higher concentrations in polluted areas than in pristine areas in the same watershed. DON concentrations show great differences in different watersheds which are disturbed by humans, but by contrast, maintain the same levels in unpolluted areas. Moreover, the local biogeochemical processes may play an important role in the reciprocal transformation of the N species [19,21]. The DON values in the Mun River Basin averaged 0.48 mg/L, higher than the DON concentrations in pristine areas. DOC was relatively lower in the pristine areas compared with the agriculturally dominated catchment. In the Mun River Basin, the clearly high concentrations of DOC (averaged 17.41 mg/L) are most likely associated with agricultural activities. The current water quality data suggest that water management should be implemented to reduce anthropogenic inputs, especially the organic material level as health-threatening environmental issues of the Mun River Basin.

## 4. Conclusions

This study reported data on C and N species contents in the Mun River, Thailand. DOC values exhibited clearly high concentrations (averaging 11.03 mg/L), at twice the level of the worldwide average value (5.35 mg/L). DON values averaged 0.48 mg/L within the range of the polluted area. DOC, NO_3_^−^, and DON exhibited spatial distributions at different sub-watersheds while NH_4_^+^ maintained the same level over the whole watershed. DON is the main form of the N pool in the Mun River, accounting for 65% of TDN. The abnormal ratios between DOC and DON in the Mun River most likely come from anthropogenic inputs. The significant correlation of DOC and DON in the Upper Mun and Lower Mun imply that they come from same source. The limited correlations between the N species (DON, NO_3_^−^, and NH_4_^+^) demonstrate that both the anthropogenic inputs and biogeochemical processes play important roles in the riverine N pool and influence the reciprocal transformation of the N species. Based on the ions Cl^−^ and NO_3_^−^, the correlation analyses indicate that there are different anthropogenic sources at different sub-watersheds that impact the water chemistry, consistent with the different origins of DOC and DON. The water quality evaluation and the data collected in rivers worldwide imply that pollution, especially organic contamination, has already threatened water quality and may turn into a potential health risk. Thus, it is essential for the local government to introduce legislation restricting the anthropogenic inputs discharged into rivers and devote more attention to ecological conservation and environment pollution control.

## Figures and Tables

**Table 1:** Pearson correlation coefficients between C, N species and physicochemical parameters in the Mun River Basin, Thailand.

Parameters	T	pH	TDS	DO	DOC	DON	NO_3_^−^	NH_4_^+^	DOC/DON	HCO_3_^−^
T	1									
pH	0.14	1								
TDS	0.36 **	0.39 **	1							
DO	−0.31 *	0.47 *	−0.20	1						
DOC	−0.15	−0.43 **	−0.20	0.03	1					
DON	0.16	0.37 **	0.42 **	−0.27 *	−0.12	1				
NO_3_^−^-N	0.17	0.24	0.22	−0.12	−0.24	0.21	1			
NH_4_^+^-N	0.01	0.07	0.00	0.03	−0.06	−0.00	0.40 **	1		
DOC/DON	−0.25	−0.49 **	−0.31 *	0.15	0.90 **	−0.39 **	−0.05	0.10	1	
HCO_3_^−^	0.28 *	0.77 **	0.63 **	0.02	−0.35 **	0.68 **	0.32 *	0.06	−0.47 **	1

* Significance at 0.05 probability level; ** Significance at 0.01 probability level. T: temperature; TDS: total dissolved solids; DO: dissolved oxygen; DOC: dissolved organic carbon; DON: dissolved organic nitrogen.

**Table 2:** Distributions of DOC and N species in the Mun River.

Dissolved Load	Upper Mun		Middle Mun		Lower Mun	
	Range	Mean Value	Range	Mean Value	Range	Mean Value
	mg/L		mg/L		mg/L	
DOC	1.71–15.84	5.10	4.10–35.77	10.46	3.47–40.08	17.41
DON	0.22–1.37	0.67	0.25–0.72	0.41	0.20–0.54	0.34
NO_3_-N	0.02–1.07	0.26	0.01–0.57	0.13	0.02–0.16	0.06
NH_4_-N	0.11–0.29	0.16	0.11–0.23	0.16	0.10–0.17	0.13

## Supplementary Materials

The following are available online at https://www.mdpi.com/1660-4601/16/4/659/s1. Table S1: The sampling locations, water parameters, and chemical compositions of Mun River water; Table S2: DOC and DON concentrations of worldwide rivers.
