# Supplementary material for: Impacts of Anthropogenic Changes on the Mun River Water: Insight from Spatio-Distributions and Relationship of C and N Species in Northeast Thailand
Source: Int J Environ Res Public Health. 2019 Feb 23;16(4):659. doi: 10.3390/ijerph16040659 (PMC6406437; doi:10.3390/ijerph16040659)
Supplement: Supplementary file 1 [file ijerph-16-00659-s001.pdf]

Table 1. The sampling locations, water parameters and chemical compositions of the Mun River **water.**

Sample Number	Location		Temperature (℃)	pH	DO (mg/L)	TDS (mg/L)	DOC (mg/L)	DON (mg/L)	NO_3^--N (mg/L)	NH_4^+-N (mg/L)	Cl^- (mg/L)	HCO_3^- (mg/L)
	Lat.North	Long.East										
					**Upper Mun**							
U1	14°30′43.49″	101°22′49.10″	24.5	7.34	7.12	16	1.71	0.25	0.47	0.11	2.17	12.03
U2	14°41′52.17″	101°24′54.65″	25.2	7.99	6.47	54	2.53	0.53	0.14	0.17	3.03	61.56
U3	14°48′08.31″	101°31′40.68″	30.4	8.42	7.94	139	3.05	0.27	0.52	0.18	12.39	148.60
U4	14°51′22.64″	101°35′34.61″	29.1	8.42	6.75	158	2.88	0.26	-	0.15	20.05	141.52
U5	14°55′57.80″	101°57′7.21″	29.3	7.67	4.60	249	4.53	0.92	0.11	0.20	45.64	183.98
U6	14°56′59.93″	101°59′12.86″	27.4	7.62	4.54	234	4.22	1.37	0.11	0.12	43.59	169.82
U7	14°58′04.99″	102°14′16.50″	30.1	7.61	5.76	136	5.31	1.04	0.12	0.19	32.54	84.91
U8	15°03′57.88″	102°24′09.24″	29.7	7.21	4.00	85	4.67	0.42	0.67	0.18	13.77	67.10
T1	14°29′38.56″	101°41′05.74″	26.3	7.69	6.03	76	4.16	0.69	0.12	0.29	6.30	67.22
T2	14°25′45.80″	102°05′13.89″	25.8	7.23	6.71	23	2.21	0.22	-	0.17	3.85	12.20
T3	14°28′17.17″	102°07′25.18″	30.5	7.93	6.91	32	3.65	0.26	-	0.15	4.75	24.77
T4	14°33′51.17″	102°10′17.17″	30.0	7.22	3.41	107	5.93	0.70	0.10	0.15	21.54	79.91
T5	14°44′42.19″	102°12′42.83″	29.1	7.40	5.11	135	15.84	1.28	-	0.17	28.32	87.03
T6	14°59′57.25″	102°49′01.66″	30.9	7.06	5.73	47	6.44	0.53	0.05	0.15	9.82	31.13
T7	15°10′51.08″	102°15′31.15″	31.1	7.55	4.70	998	7.15	0.50	0.11	0.12	603.81	120.29
T8	15°09′34.88″	102°22′07.21″	29.5	7.42	4.10	303	6.33	1.20	0.46	0.18	109.85	130.91
T9	15°13′11.76″	102°25′42.19″	30.4	7.38	3.50	332	6.15	1.01	1.07	0.14	132.32	116.75
T10	15°15′16.53″	102°31′49.68″	30.2	7.37	4.05	391	5.64	1.07	0.08	0.21	171.75	120.29
T11	15°21′54.73″	102°44′53.57″	30.4	7.14	5.09	102	5.33	0.51	0.02	0.14	36.83	38.92
T12	15°26′07.44″	103°00′45.49″	30.4	6.90	3.59	162	4.24	0.30	0.09	0.15	75.53	33.96
					**Middle Mun**							
M1	15°17′50.31″	103°12′09.77″	30.1	6.89	4.80	60	4.09	0.45	0.13	0.15	10.67	45.29
M2	15°18′00.54″	103°17′31.80″	30.1	6.70	3.28	130	17.07	0.54	0.11	0.15	60.95	33.26
M3	15°19′46.30″	103°40′57.79″	30.6	6.80	3.92	88	4.61	0.42	0.26	0.21	36.17	24.77
M4	15°17′49.28″	103°55′44.06″	30.7	6.72	3.76	93	17.36	0.72	0.01	0.12	38.29	24.77
M5	15°20′02.00″	104°02′38.37″	30.4	6.56	4.40	22	5.00	0.33	0.57	0.22	5.10	9.15
M6	15°20′22.87″	104°09′09.11″	30.0	6.76	4.08	73	4.62	0.35	0.11	0.16	29.98	19.81
M7	15°07′04.84″	104°19′07.31″	27.9	6.36	4.74	20	35.77	0.38	-	0.11	5.58	8.14
M8	15°07′56.11″	104°29′18.11″	27.7	6.80	5.50	62	22.07	0.32	-	0.12	23.15	18.30
T13	14°52′31.93″	103°22′44.13″	28.8	6.66	4.71	34	5.00	0.37	0.13	0.15	7.37	20.52
T14	15°08′29.31″	103°25′44.66″	20.3	6.67	4.24	33	4.73	0.65	0.02	0.13	6.74	19.11
T15	14°51′26.78″	103°28′36.93″	28.8	6.87	5.06	25	4.94	0.34	0.14	0.16	4.63	17.69

T16	15°02′53.91″	104°01′15.24″	29.0	6.48	4.99	21	13.22	0.41	-	0.16	5.63	10.61
T17	15°34′18.12″	103°49′11.65″	30.0	6.47	2.97	63	4.29	0.28	-	0.23	27.67	14.15
T18	15°27′40.95″	103°53′40.14″	29.7	6.80	3.91	97	4.80	0.36	0.04	0.21	40.50	26.18
T19	15°28′09.82″	104°04′07.17″	30.2	6.45	3.68	59	5.70	0.25	0.03	0.19	26.05	10.61
T20	15°00′37.55″	104°07′42.25″	28.3	6.46	4.94	19	14.09	0.39	0.01	0.17	4.89	9.15
					**Lower Mun**							
L1	15°06′40.43″	104°40′44.01″	27.3	6.50	5.19	19	15.81	0.24	-	0.11	4.99	10.61
L2	15°09′05.63″	104°43′35.46″	27.6	6.76	5.29	50	20.69	0.34	-	0.10	18.38	17.69
L3	15°12′34.16″	104°45′24.63″	28.4	7.03	5.18	86	14.94	0.32	-	0.12	31.29	31.84
L4	15°12′18.67″	104°48′49.99″	28.7	7.05	5.17	86	26.42	0.55	0.02	0.15	30.48	28.30
L5	15°14′2.34″	105°09′38.31″	27.3	6.51	4.94	16	11.36	0.28	-	0.12	3.78	5.49
L6	15°16′22.42″	105°13′12.19″	27.6	6.81	4.66	46	10.37	0.28	-	0.11	12.96	21.23
L7	15°13′36.18″	105°18′3.76″	27.7	6.52	5.49	11	17.94	0.25	-	0.15	1.74	8.54
L8	15°17′10.75″	105°20′19.87″	27.7	6.89	5.16	46	4.88	0.34	0.07	0.13	13.88	21.23
L9	15°20′08.31″	105°23′59.85″	27.0	6.68	6.80	10	33.07	0.29	-	0.12	1.64	6.10
L10	15°15′53.85″	105°26′19.91″	27.9	6.95	5.24	48	5.87	0.30	-	0.13	14.41	21.23
L11	15°15′18.73″	105°27′05.29″	28.1	6.64	5.96	9	14.33	0.28	0.16	0.17	1.90	5.49
L12	15°13′24.56″	104°51′30.59″	28.8	7.30	4.83	88	18.49	0.54	0.02	0.17	27.48	42.46
L13	15°18′37.44″	105°29′35.22″	27.2	7.11	5.73	44	23.60	0.47	0.07	0.11	13.21	24.77
L14	15°19′07.85″	105°33′10.91″	27.1	7.36	5.89	50	19.70	0.28	0.13	0.15	5.47	42.46
T21	15°00′23.02″	104°38′00.60″	27.7	6.44	5.01	18	11.11	0.24	-	0.10	4.93	9.15
T22	15°30′10.94″	104°35′43.16″	27.5	6.54	4.53	28	26.45	0.47	-	0.17	8.71	10.61
T23	15°15′55.84″	104°38′40.70″	28.4	7.26	5.02	89	10.19	0.42	-	0.10	24.12	45.99
T24	15°17′8.71″	104°44′18.28″	27.9	6.45	4.58	32	40.08	0.41	0.04	0.15	11.39	11.32
T25	15°30′14.95″	104°57′57.28″	26.7	6.52	5.05	25	19.25	0.33	0.02	0.13	8.27	10.61
T26	15°20′17.28″	105°04′30.30″	26.2	6.50	4.66	23	3.47	0.20	-	0.11	7.06	9.91
T27	15°18′24.55″	105°07′20.73″	27.0	6.79	5.08	47	17.70	0.26	-	0.11	16.36	21.23

'-' means undetected; 'T' means tributary.

Table 2. DOC and DON concentrations of worldwide rivers.

Rivers	Observations	DON or DOC mg/L (min-max)	Reference
New Jersey, East Bass River<br>Delaware (NewJersey)<br>Hudson (NewYork)<br>Altamaha (Geor-gia)<br>Savannah (Georgia)<br>Pocomoke (Maryland)<br>Choptank (Maryland),<br>Peconic (New York)<br>Rivers	9 rivers along the east coast of the United States: The New Jersey, Delaware, Hudson, Bass, Altamaha, Savana watersheds dominated by forests. Pocomoke, Choptank and Peconic Rivers are dominated by human activities	Forests dominated area：<br>0.02–0.18 (DON)<br>4.46–7.18 (DOC)<br>polluted area：<br>0.21–0.49 (DON)<br>4.45–9.01 (DOC)	[1]
Mullica River watershed	Atlantic white cedar bogs Chamaecyparis thyoides located in the Pine Barrens region of New Jersey. River waters for analyses were collected from two pristine and two polluted areas	Pristine area:<br>0.13–0.29 (DON)<br>0.19–4.85 (DOC)<br>polluted areas：<br>0.19–0.70 (DON)<br>4.46–26.4 (DOC)	[2]
Guadalquivir River	Watershed located in the central-eastern part of the province of Jaén in south-east Spain. Agriculturally dominated catchment.	6.3-19.8 (DON)	[3]
Nile River	Nile River Damietta Branch located in northern Egypt. The Nile-Damietta Branch is subject to anthropogenic inputs from agricultural run-off, domestic wastewater , industrial activities and fish cages and extensive boating activities.	0.23–0.30 (DON)<br>2.81–8.41 (DOC)	[4]
Ovens River	The watershed is located in south-eastern Australia and drains the Victorian highlands. 63 -99% of total area is forest, followed by agricultural field.	0.02–0.69 (DON)<br>0.22 -7.14 (DOC)	[5]
Brazos River	The cities of and rural areas surrounding Bryan and College Station in south-central Texas. Mainly pasture and crop agricultural field	0.60–1.90 (DON)<br>20.40 -52.50 (DOC)	[6]
Otonabee River	Watersheds of south-central Ontario, Canada. Agriculturally dominated catchment.	1.7-24.1 (DOC)	[7]
Mississippi River	The Mississippi River is the largest river in North America and the sixth largest	0.6-5.35 (DOC)	[8]

	river in terms of water discharge in the world. Contaminated by human activities.		
World rivers	Major rivers in subarctic and tropical zone, which are still unpolluted.	World average<br>5.35 (DOC)<br>0.15-1.1 (DON)	[9]

Reference

1. Wiegner, T.N.; Seitzinger, S.P.; Glibert, P.M.; Bronk, D.A. Bioavailability of dissolved organic nitrogen and carbon from nine rivers in the eastern United States. *Aquat. Microb. Ecol*. **2006**, *43*, 277–287, doi:10.3354/ame043277.
2. Wiegner, T.N.; Seitzinger, S.P. Seasonal bioavailability of dissolved organic carbon and nitrogen from pristine and polluted freshwater wetlands. *Limnol. Oceanogr.* **2004**, *49*, 1703–1712, doi:10.4319/lo.2004.49.5.1703.
3. Lorite-Herrera, M.; Hiscock, K.; Jiménez-Espinosa, R. Distribution of dissolved inorganic and organic nitrogen in river water and groundwater in an agriculturally-dominated catchment, south-East Spain. *Water. Air. Soil Pollut*. **2009**, *198*, 335–346, doi:10.1007/s11270-008-9849-y.
4. Badr, E.S.A. Spatio-temporal variability of dissolved organic nitrogen (DON), carbon (DOC), and nutrients in the Nile River, Egypt. *Environ. Monit. Assess*. **2016**, *188*, 580, doi:10.1007/s10661-016-5588-5.
5. Harris, C.W.; Rees, G.N.; Stoffels, R.J.; Pengelly, J.; Barlow, K.; Silvester, E. Longitudinal trends in concentration and composition of dissolved organic nitrogen (DON) in a largely unregulated river system. *Biogeochemistry* **2018**, *139*, 139–153, doi:10.1007/s10533-018-0462-x.
6. Aitkenhead-Peterson, J.A.; Steele, M.K.; Nahar, N.; Santhy, K. Dissolved organic carbon and nitrogen in urban and rural watersheds of south-central Texas: Land use and land management influences. *Biogeochemistry* **2009**, *96*, 119–129, doi:10.1007/s10533-009-9348-2.
7. Wilson, H.F.; Xenopoulos, M.A. Ecosystem and seasonal control of stream dissolved organic carbon along a gradient of land use. *Ecosystems* **2008**, *11*, 555–568, doi:10.1007/s10021-008-9142-3.
8. Wang, X.C.; Chen, R.F.; Gardner, G.B. Sources and transport of dissolved and particulate organic carbon in the Mississippi River estuary and adjacent coastal waters of the northern Gulf of Mexico. *Mar. Chem.* **2004**, *89*, 241–256, doi:10.1016/j.marchem.2004.02.014.
9. Meybeck, M. Carbon, nitrogen, and phosphorus transport by world rivers. *Am. J. Sci.* **1982**, *282*, 401–450, doi:10.2475/ajs.282.4.401.
